# Gap arthroplasty with disc repositioning for pediatric TMJ ankylosis secondary to intracranial condylar dislocation: A case report

**DOI:** 10.1016/j.ijscr.2025.111364

**Published:** 2025-04-25

**Authors:** Ahmed Al Qattan, Ahmed Al Hashmi, Ibrahim Naser, Paul Sambrook

**Affiliations:** a Oral and Maxillofacial National Unit, Al Nahdha Hospital, Muscat, Oman; b Oral and Maxillofacial Department, University of Adelaide Hospital, Adelaide, Australia

**Keywords:** Arthroplasty, Temporomandibular, TMJ, Intermaxillary

## Abstract

**Introduction and importance:**

A rare consequence of maxillofacial trauma of an intracranial dislocation of the mandibular condyle into the middle cranial fossa. We present a case involving the management of this dislocation, complicated by temporomandibular joint (TMJ) ankylosis.

**Case presentation:**

An 11-year-old boy was transferred to a tertiary hospital following a road traffic accident. Examination revealed a dislocation of the right mandibular condyle into the middle cranial fossa. After closed reduction by intermaxillary fixation (IMF), the patient developed TMJ ankylosis and the initial mouth opening was 8 mm due to prolonged IMF. He subsequently referred to the National Maxillofacial Unit in Muscat, Oman for expert management and treatment.

**Clinical discussion:**

A pre-operative CT scan revealed an ankylotic bone mass in the left TMJ area. The patient underwent TMJ gap arthroplasty and disc repositioning under general anesthesia. Post-operatively, the patient's mouth opening improved to 43 mm.

**Conclusion:**

Multidisciplinary team for management and close monitory such cases is crucial to prevent further surgical complications.

## Highlights

• The management of this dislocation, complicated by temporomandibular joint (TMJ) ankylosis
• After closed reduction by intermaxillary fixation (IMF), there was TMJ ankylosis and the initial mouth opening was 8 mm
• After TMJ gap arthroplasty and disc repositioning under general anesthesia. Post-operatively, the patient's mouth opening improved to 43 mm

## 1 Introduction

Ankylosis refers to the abnormal fusion or immobilization of a joint, in this case, the Temporomandibular joint dysfunction (TMJ). TMJ ankylosis can occur due to trauma, infection, inflammatory conditions, congenital or developmental disorders, neoplasm, iatrogenic causes or/and systemic factors.

Intracranial dislocations of the mandibular condyle, which can lead to ankylosis, typically result from severe trauma or certain medical conditions affecting the TMJ. When the mandibular condyle is displaced into the cranial cavity, it disrupts normal jaw function which potentially causing complications such as limited mouth opening, pain, and difficulty in chewing and speaking.

Treatment for intracranial dislocations of the mandibular condyle with resultant ankylosis often involves surgical intervention to reposition the condyle and restore joint function. Surgical options may include gap arthroplasty, condylectomy, joint reconstruction or TMJ arthroplasty. Post-surgical physical therapy and rehabilitation are essential to regain normal jaw movement and function. Early diagnosis and intervention are crucial to prevent long-term complications and restore optimal jaw function.

While traumatic dislocation of the mandibular condyle is rare, direct dislocation into the middle cranial fossa is even more uncommon, with approximately 59 reported cases in English literature [1]. The earliest cases were documented by Heindseick and Dingman in 1960, followed by Grabb in 1963 [2]. This scarcity is linked to its association with high-velocity road traffic accidents. The primary cause frequently cited was road traffic accidents (53 %), followed closely by falls and bicycle-related injuries. This dislocation was predominantly observed in children and young adults (75 %) and was more prevalent among women (69 %) than men [1,3].

Detecting intracranial dislocation of the mandibular condyle is challenging, with initial diagnosis successful in only half of the cases, while the rest are identified after initial treatments fail. Diagnostic difficulties arise from the lack of distinct signs or symptoms specific to this injury and the limited utility of conventional radiography due to the overlap of surrounding structures. Delayed mobilization after reducing the injury can result in ankylosis of the dislocated mandibular condyle within the middle cranial fossa, as observed in our case.

In this paper we are presenting a rare case of TMJ ankylosis following an intracranial dislocation of the mandibular condyle, despite successful initial reduction.

## 2 Case study

This case report details the multidisciplinary management of an 11-year-old boy who was transferred from a peripheral hospital to a tertiary hospital following a road traffic accident. The patient presented with a displaced mandibular condyle and a basal skull fracture. His symptoms included vomiting, amnesia, loss of consciousness, otorrhea, rhinorrhea, and bilateral dilated pupils with anisocoria. Examination revealed malocclusion, tenderness at the TMJ and impaired extraocular movements. Initially, a multidisciplinary team consisting of craniofacial surgery, neurosurgery, ear, nose, and throat doctor (ENT), ophthalmology and pediatrics managed the patient, before referring him to maxillofacial surgery when ankylosis developed. Informed consent was obtained from the patients for the study.

A computed tomography (CT) scan revealed multiple significant findings that includes a longitudinal fracture in the right petrous bone, a comminuted fracture in the right tympanic part of the temporal bone with hemotympanum and a bone fragment in the middle ear. Additional fractures were observed in the clivus bone, the left mandibular fossa roof causing left mandibular head dislocation, and the left squamous part of the temporal bone. Air locules were found in Cerebrospinal fluid (CSF) spaces, but no intracranial hematoma or mass effect was noted. No active intervention was undertaken by ENT and ophthalmology. However, the neurosurgery and craniofacial surgery teams performed an open reduction of the displaced left mandibular condyle, a left temporal craniotomy, repair of a dural tear, and glenoid fossa bone grafting, using a donor site from the calvaria bone through a coronal approach. The patient was then placed on rigid IMF.

**Fig. 1:**
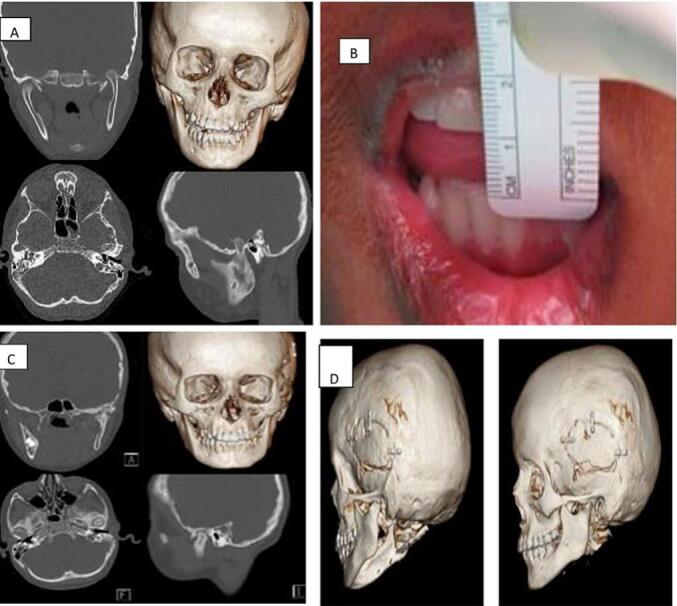

The IMF was removed after 20 days, but the patient exhibited limited mouth opening and restricted range of motion. Despite recommendations for aggressive mouth opening exercises under close monitoring, the patient's condition did not improve. He was then referred to the maxillofacial national unit hospital and arrived at our department 10 months later. Upon clinical examination, cranial nerve function was intact, but mouth opening was limited to 8 mm. A CT scan was obtained and revealed an ankylotic mass in the left TMJ region (Fig. 1).

Under general anesthesia and through a standard preauricular approach patient had release of TMJ ankylosis and repositioning of the article disc. This is our routine management protocol for pediatric TMJ ankylosis, which is the minimal removal of lateral ankylotic mass then mobilization and reduction of the original articulate disc and utilize it as the normal barrier to restore normal anatomy.

**Fig. 2:**
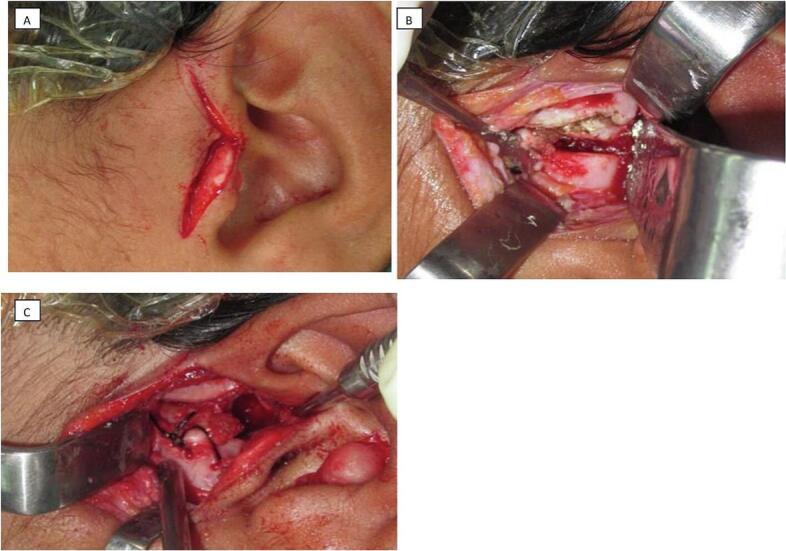

A gap arthroplasty was performed on the left TMJ under general anesthesia. Access was gained through a pre-auricular incision followed by dissection to connect the temporal and zygomatic root portions. Subperiosteal and subfascial dissection exposed the ankylosis limits, with careful protection of facial nerve branches. Piezosurgery was used to remove 2.1 cm of the ankylotic mass, avoiding vascular injury. The joint cavity was irrigated, irregular edges were smoothed with Piezosurgery and the displaced disc, located anterior-medially, was repositioned using non-resorbable sutures (Fig. 2). The mouth opening achieved 43 mm intraoperative. The patient was on close monitoring follow-up along with physiotherapist team. He was on aggressive hand physiotherapy and was maintained with him by using TheraBite. He maintained the mouth opening of 41 mm for the two years with no sign of any restrictions. A,ental pantomogram radiograph was taken postoperatively, and a 3D cone beam computed tomography scan was performed during follow-up, showing the maintenance of a clear gap in condylar area, significant ramus remodeling and proper alignment of the mandibular structures. The work has been reported in line with the SCARE criteria. [4]

## 3 Discussion

Unlike injuries to the condylar head and neck, condylar intrusion injuries are exceedingly uncommon because the fracture at condylar base is a protective mechanism to prevent a more serious intracranial injury. The cranium and its internal components are protected from penetration due to the structural vulnerability of the condylar neck. This area is particularly prone to fracturing upon impact to the mandible, thus absorbing forces that might otherwise lead to brain injury [5].

The type of condylar injury depends on the direction of the forces applied to the lower jaw, TMJ, and the anatomy of the skull base and jaw position. Sometimes, the condyle can extend into the cranial cavity through the glenoid fossa. While the lateral walls of this fossa are robustly constructed, the roof is made up of a delicate bony layer that separates the condyle from the middle cranial fossa [6].

Imaging findings in our case reveal significant CT scan of the head reveals a longitudinal fracture of the right petrous bone and a comminuted fracture of the tympanic part of the temporal bone, situated at the posterior margin of the right mandibular fossa. Hemotympanum and a small bone fragment within the middle ear cavity are also observed. The ossicle chain appears normal without any evident disruption. Multiple air locules are observed within various CSF spaces, including the temporal horn of the left lateral ventricle, anterior horn of the right lateral ventricle, prepontine cistern, falx cerebri, sella turcica, and around the proximal cervical part of the spinal cord. There is no indication of intracranial hematoma or hemorrhage, nor any evidence of mass effect or midline shift.

Additional factors that have been documented to elevate the chances of superior condylar dislocation include heightened pneumatization of the temporal bone and absence of posterior occlusion or open mouth position during the traumatic event [7]. CT and magnetic resonance imaging of the TMJ offer enhanced reliability in detecting intracranial dislocation of the mandibular condyle due to the clearer visualization achieved by eliminating overlapping structures, as discussed in Barron et al. According to their findings, a CT scan played a crucial role in accurately diagnosing the condition in 17 out of 48 patients [8].

Delayed mobilization of this injury may result in mandibular condyle ankylosis within the glenoid fossa, leading to complications such as functional limitations, disruption of mandibular growth, and dentofacial asymmetry. The initial surgery was successful to reduce the intracranially dislocated mandible and managed to restore normal barrier between the cranium and the mandible. However, early mobilization and reduction of the dislocated articulate disc could have prevented the TMJ ankyskosis.

A slender-necked condyle disperses the upward forces, lessening the risk of skull and brain injury. In contrast, younger individuals typically possess a more rounded condyle with a thicker neck, which is less prone to fracture but more likely to dislocate instead [9]. Other speculated factors for the dislocation may involve the absence of rear occlusion and greater pneumatization of the temporal bone [10]. Another significant discovery from reviewing literature indicates that in most cases, the condyle remained intact when dislocated into the middle cranial fossa. The fracturing of the involved condyle was uncommon, occurring in only 6 out of 43 instances of intracranial dislocation [11]. This mechanical problem of mouth opening obstruction cannot be resolved through the use of muscle relaxants or neuromuscular blocking agents [3]. Initially, treatment involved exposing the dislocated condyle and removing the fragment within the middle cranial fossa [[12], [13], [14]], or performing a subcondylar osteotomy, leaving the dislocated head of the condyle in place to create a barrier between the middle cranial fossa and glenoid cavity [2].

However, in both cases, the dislocation was detected at a late stage. The temporalis fascia and muscle were utilized as an interpositional substance between the fresh glenoid fossa and articular surface. The placement of this material could potentially deter degenerative alterations and hinder the occurrence of ankylosis [15].

Closed reduction was suggested as the preferred treatment for children still growing to avoid interfering with the natural growth of the jaw [3,16]. However, closed reduction was not favored for fracture-dislocations due to the challenges associated with realigning the bones so other surgical options such as gap arthroplasty, interpositional arthroplasty with or without costochondral graft, condylotomy or total joint reconstruction was discussed. The Kaban protocol for managing temporomandibular joint ankylosis was preferred in this case. This approach involved aggressive resection of the ankylosed joint, the use of a costochondral graft (CCG), placement of interpositional material, early mobilization with physical therapy and long-term follow-up [17].

In situations where there was a risk of serious intracranial lesions during manipulation, arthroplasty procedures were performed. While central condylar dislocations in the middle cranial fossa are uncommon, they might coincide with short, rounded condylar necks. Detecting these dislocations early allows for successful treatment through reduction under anesthesia alone. Timely identification also minimizes the necessity for extensive surgical interventions. Despite potential damage to the disc during injury, the condyle undergoes remodeling, aiding in maintaining functionality and decreasing the risk of ankylosis.

## 4 Conclusion

In conclusion, the delayed mobilization of the intracranial dislocations of the mandibular condyle, especially pediatric patients, can represent a complex clinical challenge particularly when complicated by delayed mobilization leading to ankylosis. Careful evaluation and management are crucial. A thorough understanding of the underlying pathophysiology, combined with a multidisciplinary treatment approach, is essential for achieving optimal outcomes and enhancing the quality of life for affected individuals.

## Author contribution

Ahmed Al Qattan: Data collection and writing the paper

Ahmed Al Hashmi: Writing paper and operating surgeon

Ibrahim Naser: Concept development

Paul Sambrook: Reviewing the paper

## Consent

Written informed consent was obtained from the patient's parents for publication and any accompanying images. A copy of the written consent is available for review by the Editor-in-Chief of this journal on request.

## Ethical approval

Ethical approval for this study is approved by Sultante of Oman ministry of health on 8th June 2024.

Ethical approval for this study (Unraveling the Enigma: Intracranial Dislocations and the Road to Mandibular Ankylosis) was provided by the Ethical Committee Sultante of Oman ministry of health on 8th June 2024.

## Guarantor

Ahmed Al Qattan

Ahmed Al Hashmi

Ibrahim Naser

Paul Sambrook

## Research registration number

Nil

## Funding

Nil

## Conflict of interest statement

No conflict of interest.
